# Conditional Probability Joint Extraction of Nested Biomedical Events: Design of a Unified Extraction Framework Based on Neural Networks

**DOI:** 10.2196/37804

**Published:** 2022-06-07

**Authors:** Yan Wang, Jian Wang, Huiyi Lu, Bing Xu, Yijia Zhang, Santosh Kumar Banbhrani, Hongfei Lin

**Affiliations:** 1 School of Computer Science and Technology Dalian University of Technology Dalian China; 2 Department of Pharmacy The Second Affiliated Hospital of Dalian Medical University Dalian China; 3 School of Information Science and Technology Dalian Maritime University Dalian China

**Keywords:** nested biomedical event, joint extraction, graph convolutional network, GCN, Dice loss, syntactic structure

## Abstract

**Background:**

Event extraction is essential for natural language processing. In the biomedical field, the nested event phenomenon (event A as a participating role of event B) makes extracting this event more difficult than extracting a single event. Therefore, the performance of nested biomedical events is always underwhelming. In addition, previous works relied on a pipeline to build an event extraction model, which ignored the dependence between trigger recognition and event argument detection tasks and produced significant cascading errors.

**Objective:**

This study aims to design a unified framework to jointly train biomedical event triggers and arguments and improve the performance of extracting nested biomedical events.

**Methods:**

We proposed an end-to-end joint extraction model that considers the probability distribution of triggers to alleviate cascading errors. Moreover, we integrated the syntactic structure into an attention-based gate graph convolutional network to capture potential interrelations between triggers and related entities, which improved the performance of extracting nested biomedical events.

**Results:**

The experimental results demonstrated that our proposed method achieved the best F1 score on the multilevel event extraction biomedical event extraction corpus and achieved a favorable performance on the biomedical natural language processing shared task 2011 Genia event corpus.

**Conclusions:**

Our conditional probability joint extraction model is good at extracting nested biomedical events because of the joint extraction mechanism and the syntax graph structure. Moreover, as our model did not rely on external knowledge and specific feature engineering, it had a particular generalization performance.

## Introduction

### Background

In recent years, event extraction research has attracted wide attention, especially in biomedical event extraction, which is critical for understanding the biomolecular interactions described in the scientific corpus. Events are important concepts in the field of information extraction. However, researchers have different definitions of events, based on different research purposes and perspectives. In the general domain, an event is a specific thing that describes a state change involving different participants, such as the evaluation of automatic content extraction, in which 8 categories and 33 subcategories of events are defined in a hierarchical structure, and each type of event contains a different semantic role. In the biomedical field, McDonald et al [1] defined event extraction as multirelationship extraction, the purpose of which was to extract semantic role information between different entities in an event. For example, the biomedical natural language processing (BioNLP) evaluation task defined 9 different categories of biochemical events. Each event included an event trigger and at least one event argument, and the different event types had different semantic roles. Unlike the events in automatic content extraction, biomedical events may have nested event phenomena.

**Figure 1:**
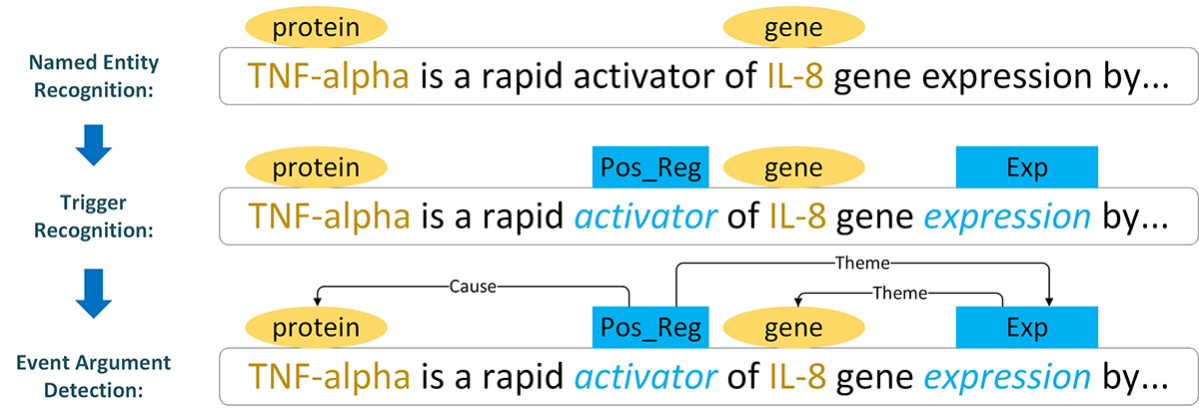
Basic progress of biomedical event extraction, where yellow boxes represent the type of entity and the blue boxes represent the type of trigger. Theme and cause represent the relationship between participant and event, namely, argument detection. IL-8: interleukin 8; TNF-alpha: tumor necrosis factor.

To clearly describe the progress of biomedical event extraction, we defined 4 concepts for biomedical events, as shown in Figure 1 and Textbox 1.

**Concepts for biomedical events.**

**Event type**

The semantic type of different events

**Event description**

A complete sentence or clause in the text that specifically describes at least one event

**Event trigger**

A word or phrase representing the occurrence of an event in the event description; usually of a *verb* or *nonverb* nature, and its category is event type; it should be noted that each event has only 1 event trigger.

**Event argument**

The event participants describe the different semantic roles in the event, whose type represents the relationship between the event and related participants; in the biomedical event system, there are 6 different semantic roles, where “theme” and “cause” are core arguments.

The task of event extraction comprises 3 subtasks: named entity recognition, trigger recognition, and event argument detection. Previous studies have relied on pipeline methods [2-5] to extract biomedical events. For example, given the event description (a sentence) shown in Figure 1, the event extraction system can find 2 entities (“TNF-alpha” and “IL-8”) in this sentence at the named entity recognition step. After recognizing triggers, it can identify a *positive regulation* (“Pos_Reg”) event mention triggered by a word *activator* and an *expression* (“Exp”) event mention triggered by a word *expression*. On the basis of the recognized entities and triggers, the system detects arguments and associates them with the related event triggers. Thus, the entity “TNF-alpha” is a participant in the *positive regulation* event, and the entity “IL-8” is a participant in the *expression* event. As the result of the previous step is the input of the subsequent step, the pipeline methods probably introduce cascading errors if the precision of the previous step is biased.

As the syntactic dependency tree enriches the feature representation, previous studies tended to use syntactic relations to improve the performance of event extraction. For example, Kilicoglu et al [2] leveraged external tools to segment sentences, annotate parts of speech (POS), and parse syntactic dependency. Then, they joined these features to extract biomedical events using a dictionary and rules. Björne et al [4] transferred the syntactic relations to the path embeddings, then combined them with word embeddings, POS embeddings, entity embeddings, distance embeddings, and relative position embeddings to feed into the convolutional neural network (CNN) model to extract biomedical events. However, the previous studies only adopted syntactic relations as the external features and ignored the interrelations between triggers and related entities obtained from the syntactic dependency tree, which improved the performance of extracting simple events but not nested events.

In this study, we mainly used the multilevel event extraction (MLEE) corpus [6] and the BioNLP shared task (BioNLP-ST) 2011 Genia event (GE) corpus [7] to evaluate our method. There is some explanation regarding the MLEE extending event extraction methods to the biomedical information field and covering all levels of biological tissue from molecules to entire organisms. The MLEE label scheme is the same as the BioNLP event system but has more abundant event types: 4 major categories (anatomical, molecular, general, and planned) and 19 subcategories. The specific information is shown in Table 1.

**Table 1:** Primary event types and argument roles in the multilevel event extraction corpus (N=6827).

Event and subevent types			Core arguments		Values, n (%)
**Anatomical**					
	Cell proliferation	Theme (entity)		133 (2.42)	
	Development	Theme (entity)		316 (4.81)	
	Blood vessel development	Theme (entity)		855 (12.91)	
	Growth	Theme (entity)		469 (2.65)	
	Death	Theme (entity)		97 (1.53)	
	Breakdown	Theme (entity)		69 (1.1)	
	Remodeling	Theme (entity)		33 (0.45)	
**Molecular**					
	Synthesis	Theme (entity)		17 (0.3)	
	Gene expression	Theme (entity)		435 (6.66)	
	Transcription	Theme (entity)		37 (0.61)	
	Catabolism	Theme (entity)		26 (0.39)	
	Phosphorylation	Theme (entity)		33 (0.5)	
	Dephosphorylation	Theme (entity)		6 (0.09)	
**General**					
	Localization	Theme (entity)		450 (6.87)	
	Binding	Theme (entity)		187 (2.92)	
	Regulation	Theme (entity or event) and cause (entity or event)		773 (11.81)	
	Positive regulation	Theme (entity or event) and cause (entity or event)		1327 (20.33)	
	Negative regulation	Theme (entity or event) and cause (entity or event)		921 (14.08)	
**Planned**					
	Planned process	Theme (entity or event)		643 (9.9)	

To abate the impact of cascading errors, we propose an end-to-end conditional probability joint extraction (CPJE) method that can effectively transmit trigger distribution information to the event argument detection task. To capture the interrelations between triggers and related entities and improve the performance of extracting nested biomedical events, we integrated the syntactic dependency tree into an attention-based gate graph convolutional network (GCN), which can capture the flow direction of the key information. The contributions of this study are as follows:

1. We propose an end-to-end CPJE framework, CPJE, which effectively leverages trigger distribution information to enhance the performance of event argument detection and weakens cascading errors in the overall event extraction process.
2. We used the syntactic dependency tree to capture the interrelations between triggers and related entities and integrated the tree into an attention-based gate GCN to extract nested biomedical events.
3. We obtained state-of-the-art performance on the MLEE and BioNLP-ST 2011 GE corpora for extracting nested biomedical events.

We summarize the current frameworks for event extraction tasks in the *Related Works* section. We introduce our framework in the *Methods* section. We display the overall performance in the *Results* section. We present the ablation study, visualization, and case study in the *Discussion* section. We summarize this work and discuss future research directions in the *Conclusions* section.

### Related Works

The biomedical event extraction problem is similar to general domain event extraction and entity relationship extraction; therefore, we have many theoretical foundations and experimental methods that can be used for reference.

#### Entity Relationship Extraction

Biomedical events can be regarded as complex relationship extraction tasks, and relationship extraction methods have achieved excellent results in various fields. Therefore, we studied some relationship extraction methods to help conceive the construction of event extraction models. With the development of deep learning, an increasing number of researchers have used deep learning algorithms to achieve the joint extraction of entity relationships [8]. To solve the problem of a sparse number of labeled samples, distant supervision methods have been applied to the relationship extraction task [9]. Deep reinforcement learning (RL) algorithms have also been applied to the relationship extraction task to solve noisy data samples [10]. In addition, with the widespread application of graph neural networks (GNNs), GCNs have been used in certain relation-extraction tasks [11,12].

#### General Domain Event Extraction

In general, news event extraction is a research hot spot. Some methods have improved the performance of event extraction by studying feature engineering. Sentence-level feature extraction included combinational features of triggers and event arguments [13] or combinational features of triggers and entity relationships [14]. Document-level feature extraction included common information event extraction from multiple documents [15] and joint event argument extraction based on latent-variable semi-Markov conditional random fields [16]. Others have also used deep learning to reduce feature engineering, which improves a model’s generalization ability and extraction performance; for example, learning context-dependency information with recurrent neural networks [17], detecting events with nonconsecutive CNNs [18], and obtaining syntactic structure information with GCNs [19]. All these methods have laid a better foundation for the extraction of biomedical events.

#### Biomedical Event Extraction

Extracting biomedical events is one of the BioNLP-STs [7,20,21]. Previous studies mainly explored human-engineered features based on a support vector machine model [22-25]. Owing to error transmission in the pipeline approach, Riedel et al [26] developed a joint model with dual decomposition, and Venugopal et al [27] leveraged Markov logic networks for joint inference. Recently, most studies have observed remarkable benefits of neural models. For example, some have started to add POS tags and syntactic parsing with different neural models [28], improved the biomedical event extraction model using semisupervised frameworks [29], attempted to use attention mechanisms to obtain the semantic relationship of biomedical texts [5], and used distributed representations to obtain context embedding [3,4,30,31]. To incorporate more information from the biomedical knowledge base (KB), Zhao et al [32] leveraged a RL framework to extract biomedical events with representations from external biomedical KBs. Li et al [33] fused gene ontology into tree long short-term memory (LSTM) models with distributional representations. Huang et al [34] used a GNN to hierarchically emulate 2 knowledge-based views from the Unified Medical Language System with conceptual and semantic inference paths. Trieu et al [35] used multiple overlapping, directed, acyclic graph structures to jointly extract biomedical entities, triggers, roles, and events. Zhao et al [36] combined a dependency-based GCN with a hypergraph to jointly extract biomedical events. Ramponi et al [37] proposed a joint end-to-end framework that regards biomedical event extraction as sequence labeling with a multilabel aware encoding strategy.

Compared with these methods, our approach joint extracts the biomedical events with a probability distribution of triggers, which alleviates the cascading errors introduced by the pipeline methods. Moreover, considering the potential interrelations between triggers and related entities, our approach integrates the syntactic structure into an attention-based gate GCN to capture the flow direction of key information, which greatly improves the extraction performance for nested biomedical events. It is important to mention that our approach does not require any external resources to assist the biomedical event extraction task.

## Methods

### Overview

This section illustrates the proposed CPJE model. Let *W*={*w*_1_,*w*_2_,...,*w*_n_} be a sentence of length *n*, where *w*_i_ is the *i*th word in a sentence. Similarly, *E*={*e*_1_,*e*_2_,...,*e*_k_} is a set of entities mentioned in a sentence, where *k* is the number of entities. As the trigger may comprise multiple tokens, we used the BIO tag scheme to annotate the trigger type of each token in the sentence. When we obtained the corresponding event trigger in the sentence, we used this information to predict the corresponding event arguments.

**Figure 2:**
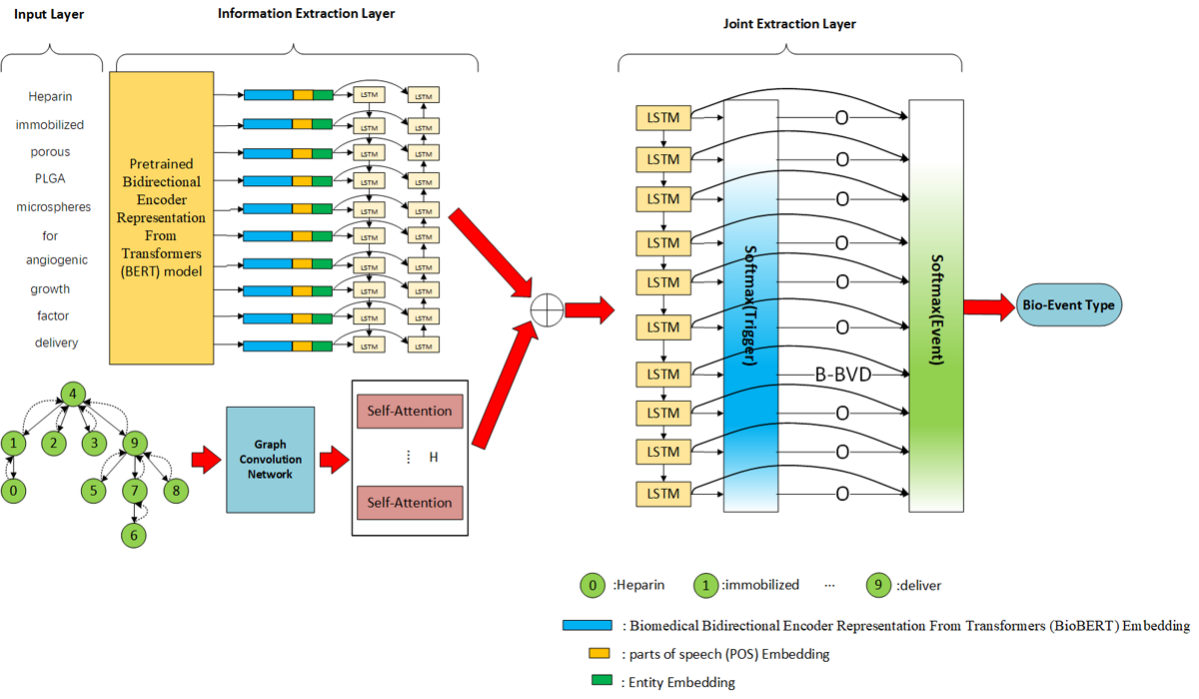
The architecture of the conditional probability joint extraction framework, where numbers 0 to 9 represent each word in the sentence, the blue bar represents BioBERT embedding, the yellow bar represents POS-tagging embedding, and the green bar represents entity embedding. BERT: Bidirectional Encoder Representation From Transformers; BioBERT: Biomedical Bidirectional Encoder Representation From Transformers; B-BVD: B-blood vessel development; LSTM: long short-term memory; POS: parts of speech.

As shown in Figure 2, our CPJE model mainly includes 3 layers: an input layer, an information extraction layer, and a joint extraction layer. The input layer converts unstructured text information (such as word sequences, syntactic structure trees, POS label representations, and entity label information) into a structured discrete representation and inputs it into the next layer. The information extraction layer converts discrete information into continuous feature representations, which deeply extracts the semantic and dependence information in a sentence. The joint extraction layer parses the previous fusion information and sends the parsed information into the trigger softmax classifier and event softmax classifier to jointly extract biomedical events.

### Information Extraction Layer

This is not explained in detail as the input layer was too superficial (only converting the text into a sequence of numbers). Each module of the information extraction layer is presented in the following sections.

#### Word Representation

In the word representation module, to improve the representation capability of the initial features, each word *w*_i_ in the sentence is transformed to a real-valued vector *x*_i_ by concatenating the embeddings described in the following sections.

#### Biomedical Bidirectional Encoder Representation From Transformers Embedding

We used the Biomedical Bidirectional Encoder Representation from Transformers (BioBERT) pretraining model [38] to obtain the dynamic semantic representation of the word *w*_i_. BioBERT embedding comprises token embedding, segment embedding, and position embedding, which is encoded as a consequence by a multilayer bidirectional transformer. Thus, it includes rich semantic and positional information. Furthermore, it can solve the polysemy problem of words. We define *a*_i_ as the word vector representation of the word *w*_i_.

#### POS-Tagging Embedding

We used a randomly initialized POS-tagging embedding table to obtain each POS-tagging vector. We defined *b*_i_ as the POS-tagging vector representation of the word *w*_i_.

#### Entity Label Embedding

Similar to the POS-tagging embedding, we used the BIO label scheme to annotate the entities mentioned in the sentence and convert the entity type label into a real-value vector by consulting the embedding table. We defined *c*_i_ as the entity vector representation of the word *w*_i_.

The transformation from the token *w*_i_ to the vector *x*_i_ converts the input sentence *W* into a sequence of real-valued vectors *X*={*x*_1_,*x*_2_,...,*x*_n_}, 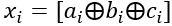, where 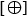 is the concatenation operation, *x*_i_ is the μ dimension (ie, the sum of the dimensions of *a*_i_, *b*_i_, and *c*_i_), and 
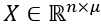
. *X* is fed into the subsequent blocks to obtain more valuable information for extracting biomedical events.

#### Bidirectional LSTM

To obtain the context information of the input text and avoid the gradient explosion problem caused by long texts, we chose the classic bidirectional LSTM (BiLSTM) structure to extract the context features of the word representations.

We fed the word representation sequence *X*={*x*_1_,*x*_2_,...,*x*_n_} into BiLSTM to obtain the forward hidden unit *h*_t_^f^ and the backward hidden unit *h*_t_^b^ with φ dimension in time *t* according to equation 1. We represented all the hidden states of the forward LSTM and backward LSTM as 
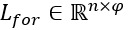
 and 
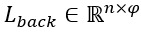
, respectively, where *n* is the number of LSTM hidden units:



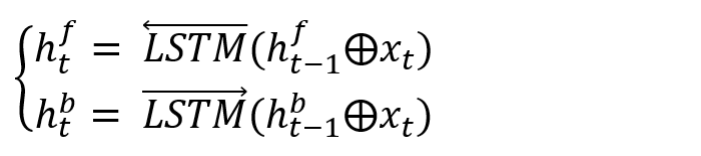


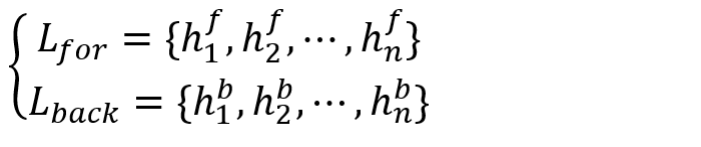



Finally, we concatenated these 2 matrices to obtain the context representation 
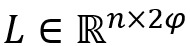
 of BiLSTM:



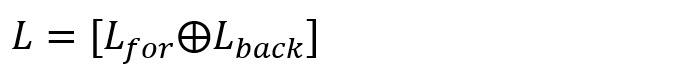



#### Gate GCN

To obtain the syntactic dependence in a sentence, we reference the method proposed by Liu et al [19] to apply a gate GCN model to analyze the sentence-dependent features. We considered an undirected graph G=(*V*, ε) as a syntactic dependency tree for the sentence *W*, where *V* is the set of nodes and ε is the set of edges. Defining 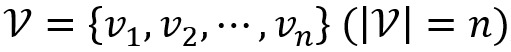, *v*_i_ represents each word *w*_i_ of sentence *W*, and each edge 
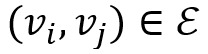
 represents a directed syntactic arc from word *w*_i_ to word *w*_j_, with dependency type *Re*. In addition, for the sake of moving information along the direction, we add the corresponding reversed edge (*v*_w_, *v*_i_) with dependency type *Re′* and self-loops (*v*_i_, *v*_i_) for any node *v*_i_. According to statistics, we used the Stanford Parser [39] to obtain approximately 50 different kinds of syntactic dependency. To facilitate the GCN internal calculation, we only considered the direction of information flow and simplified the original dependency into 3 forms, as shown in equation 4:



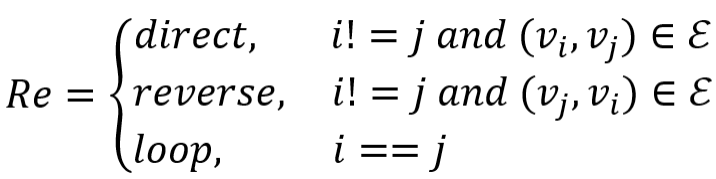



For node 
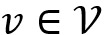
, we can use the hidden vector *h*_v_^(j)^ in the *j*th gate GCN layer to compute the hidden vector *h*_v_^(j+1)^ of the next layer:



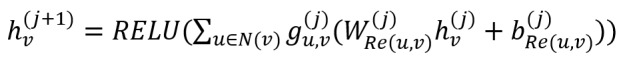



where *Re*(*u*,*v*) is the dependency type between nodes *u* and *v*, *W_Re(u,v)_*^(j)^ and *b_Re(u,v)_*^(j)^ are the weight matrix and bias, respectively. *N* (*v*) is the set of neighbors of node *v*, including *V*. The weight of edge (*u*, *v*) is *g_u,v_*^(j)^, which applies the gate to the edge to indicate the importance of the edge, as shown in equation 6:



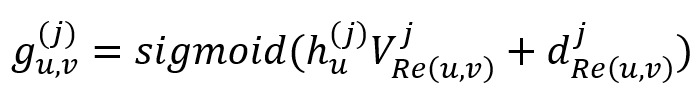



Here, *V_Re(u,v)_*^j^ and *d_Re(u,v)_*^j^ are the gate weight matrix and bias, respectively. We used BioBERT embedding *A*={*a*_1_,*a*_2_,...,*a*_n_} to initialize the input of the first GCN layer. Stacking *k* GCN layers can obtain a syntactic information matrix 
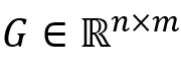
, where *m* is the dimension of node *v*_i_ with the same dimension of *a*_i_.

#### Multi-Head Attention

As shown in Figure 2, multi-head attention [40] comprises *H* self-attentions, which can thoroughly learn the similarity between nodes and calculate the importance of each node so that the model can focus on more critical node features. Let *W*_i_^Q^, *W*_i_^K^, and *W*_i_^V^ be the *i*th initialized weight matrix of *Q*, *K,* and *V*, known by equation 7:



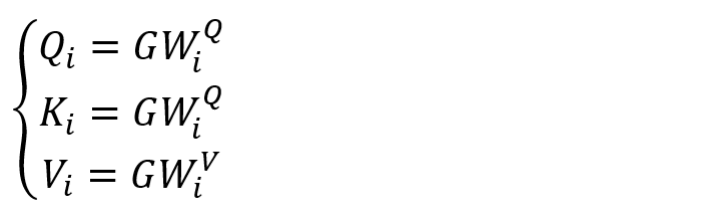



Here, 
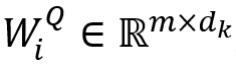
, 
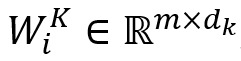
, 
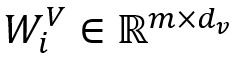
, and *d*_k_=*d*_v_=*m*/*H*.

We calculated the scoring matrix of the *i*th head according to equation 8. After concatenating *H* heads, we used equation 9 to obtain the attention output matrix *M*. 
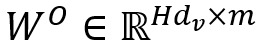
 is the linear transformation matrix:



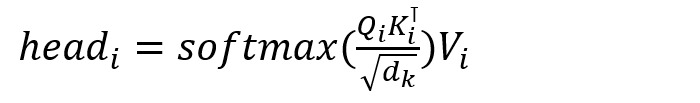


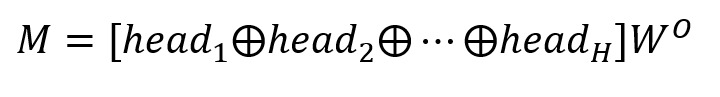



### Joint Extraction Layer

#### Tagger

The tagger comprises a unidirectional LSTM that takes the context representation given by BiLSTM as the input and the syntactic dependency representation generated by the attention GCN module to parse the information of the previous layer. Let 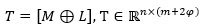. After the tagger module, we obtained the output matrix *O*, which was sent to the conditional probability extraction module.

#### Conditional Probability Extraction

Most joint extraction models input the same source information into different subtask classifiers simultaneously to achieve information sharing, as shown in equation 10, where 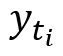 is the output of the trigger in time step *i* and 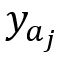 is the output of the argument in step *j*.



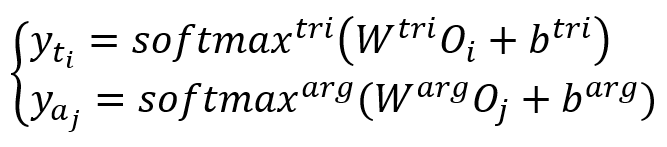



However, when the occurrence frequency of 2 subtasks in the same data set varies significantly, the model easily focuses on high-frequency subtasks and ignores low-frequency subtasks. Similar to the biomedical event extraction task, for the trigger recognition and event argument detection subtasks, each event trigger (ie, biomedical event) may contain 0, 1, or 2 participating elements, and the participating element may also be another event; therefore, the contribution of the trigger recognition task will be greater than that of the event argument detection task. To alleviate the abovementioned problems and reduce the cascading errors between these 2 subtasks, we combined the softmax output after trigger recognition and the source information to extract the trigger vector *Tr*_i_ and event argument vector *Can*_j_ according to the location of triggers and candidate arguments. Finally, by aggregating and inputting them into the event extraction classifier and learning the distribution features of the trigger label, our model directly achieved biomedical event extraction without postprocessing.



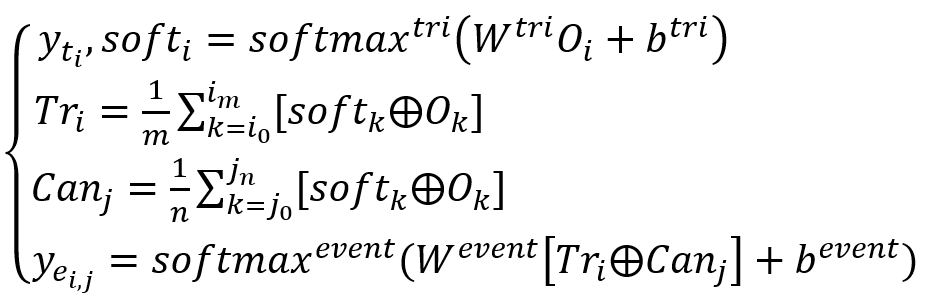



Here, *W^tri^* and *b^tri^* are the weight matrix and bias for trigger recognition, separately. The probability output of the trigger softmax of the *k*th word is *soft*_k_. *W^event^* and *b^event^* are the weight matrix and bias for event extraction, separately. The number of words of the *i*th trigger and the *j*th candidate argument are *i*_m_ and *j*_n_, separately. *O*_k_ is the source information vector of the *k*th word.

Comparing equation 10 with equation 11, we found that it only realizes the joint extraction of triggers and event arguments using equation 10; therefore, it needs postprocessing to seek out the tuple of events. However, owing to the aggregation of trigger distribution information, we can discover which event argument belongs to the trigger of step *t* using equation 11.

### Joint Dice Loss

Owing to the sparse data of the biomedical event corpus and the imbalance between positive and negative examples, the cross-entropy or negative log-likelihood loss function causes a large discrepancy between precision and recall. To alleviate this problem, we propose using a joint weight self-adjusting Dice loss function [41], as follows:



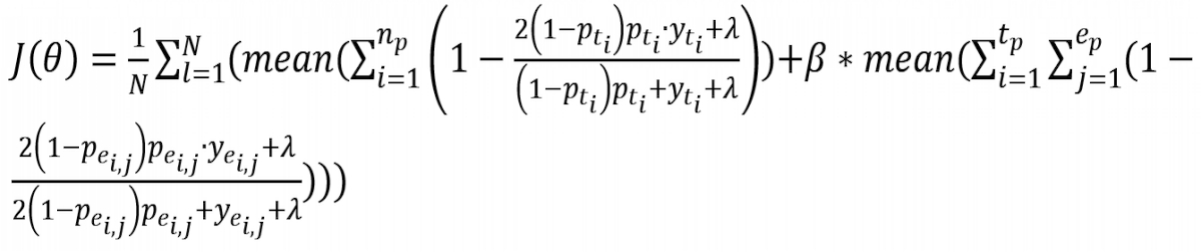



Here, *N* is the number of sentences in the corpus; *n*_p_, *t*_p_, and *e*_p_ are the number of tokens, extracted trigger candidates, and arguments of the *l*th sentence, λ is for smoothing purposes, β is a hyperparameter to adjust the loss, and θ is the model’s parameters that should be trained.

### Training

The CPJE model was trained using several epochs. In each epoch, we divided the training set into batches, each containing a list of sentences and each sentence containing a set of tokens of variable lengths. One batch was in progress at a time step.

For each batch, we first ran the information extraction layer to generate the context representation 
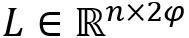
 and the attention representation with syntactic information 
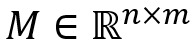
. Then, we combined *L* and *M* as the input of LSTM to generate source information *O*. In the end, we ran the joint extraction layer to compute gradients for overall network output (triggers and events). After that, we back propagated the errors from the output to the input through CPJE and updated all the network parameters. The overall procedure of the CPJE model is summarized in Textbox 2.

**The training procedure of the conditional probability joint extraction model.**

**Input**

1. Sequence of tokens {*w*_1_,...,*w*_n_} along with corresponding event labels
2. Set of edges {*e*_12_,...,*e*_ij_,...,*e*_mn_} for each corresponding token

**Output**

All parameters in the conditional probability joint extraction model

1. For each epoch do
2. For each epoch do
3. Generate *L* and *M* by information extraction layer via equations 3 and 9
4. Concatenate *L* and *M* as *T*
5. Generate the source information *O*={*o*_1_,...,*o*_n_} by long short-term memory
6. Compute the trigger scores *y*_t_ and the trigger softmax probability *soft* by the “SoftMax Trigger” block in the joint extraction layer via the first equation in equation 11
7. Fuse *O* and *soft* via the second and third equations in equation 11
8. Compute the event scores *y*_t_. by the “SoftMax Event” block in the joint extraction layer via the fourth equation in equation 11
9. Update the parameters by the back propagation algorithm
10. End for
11. End for

### Data

Our experiments were conducted mainly on the MLEE corpus [6], as shown in Table 2, which has 4 categories containing 19 predefined trigger subcategories. There are 262 documents with 56,588 words in total, with 8291 entities and 6677 events. From Table 2, we note that the number of anatomical-level events is higher than the number of molecular-level and planned-level events, although general biomedical events dominate overall. Overall, 18% (1202/6677) of the total events involved either direct or indirect arguments at both the molecular and anatomical levels. From Table 1, we find that the arguments of regulation, positive regulation, negative regulation, and planned process events may not be only entities but also other events; therefore, these events are nested events, which account for approximately 54.87% (3664/6677) of all events.

**Table 2:** The multilevel event extraction statistical information.

Item			Training, n (%)		Development, n (%)		Test, n (%)		Total, N
Document			131 (50)		44 (16.8)		87 (33.2)		262
Sentence			1271 (48.73)		457 (17.52)		880 (33.74)		2608
Word			27,875 (49.26)		9610 (16.98)		19,103 (33.76)		56,588
Entity			4147 (50.02)		1431 (17.26)		2713 (32.72)		8291
**Event**			3296 (49.36)		1175 (17.6)		2206 (33.04)		6677
	Anatomical	810 (48.36)		269 (16.06)		596 (35.58)		1675	
	Molecular	340 (48.2)		125 (17.7)		240 (34.0)		705	
	General	1851 (50.66)		627 (17.16)		1176 (32.18)		3654	
	Planned	295 (45.9)		154 (24.0)		194 (30.2)		643	

In addition, we verified our experiment using the BioNLP-ST 2011 GE corpus [7]. As shown in Table 3, the BioNLP-ST 2011 GE corpus defines 9 biomedical event types. It is noted that a *binding* event probably requires >1 protein entity as its theme argument, and a *regulation* event is likely to require a protein or an event as its theme argument and needs a protein or an event as its cause argument. There were 37.20% (9288/24,967) of events (regulation, positive regulation, and negative regulation) that led to a nested structure.

**Table 3:** The primary event types and core argument roles in the BioNLP-STa 2011 GEb corpus and the important statistical information of the GE corpus.

Event types and BioNLP-ST 2011 GE items		Core arguments	Values, N
**Event type**			
	Gene expression	Theme (protein)	N/A^c^
	Transcription	Theme (protein)	N/A
	Protein catabolism	Theme (protein)	N/A
	Phosphorylation	Theme (protein)	N/A
	Localization	Theme (protein)	N/A
	Binding	Theme (protein)^d^	N/A
	Regulation	Theme (protein or event) and cause (protein or event)	N/A
	Positive regulation	Theme (protein or event) and cause (protein or event)	N/A
	Negative regulation	Theme (protein or event) and cause (protein or event)	N/A
**BioNLP-ST 2011 GE corpus statistics**			
	Document	N/A	1224
	Word	N/A	348,908
	Entity	N/A	21,616
	Event	N/A	24,967

^a^BioNLP-ST: BioNLP shared task.

^b^GE: Genia event.

^c^N/A: not applicable.

^d^Represents the number of arguments >1.

### Hyperparameter Setting

For the hyperparameter settings of our experiment, we used 768 dimensions for the BioBERT embeddings and set 64 dimensions for the POS-tagging and entity label embeddings. We applied a 1-layer BiLSTM with 128 hidden units and used a 2-layer GCN and 2-head self-attention for our model. The dropout rate was 0.3, the learning rate was 0.01, and the optimization function was stochastic gradient descent (SGD). The training of our CPJE model was based on the operating system of Ubuntu 20.04, using PyTorch (version 1.9.0) and Python (version 3.8.8). The graphics processing unit was an NVIDIA TITAN Xp with 12 GB of memory.

## Results

### Overall Performance on MLEE

We compare our performance with the baselines shown in Textbox 3.

**Baselines for performance.**

**EventMine**

Pyysalo et al [6] applied a pipeline-based event extraction system, mainly relying on support vector machine classifiers to implement trigger recognition and event extraction.

**Semisupervised learning**

This is a semisupervised learning framework proposed by Zhou et al [30], which can use unannotated data to extract biomedical events.

**Convolutional neural network**

Wang et al [3] used convolutional neural networks and multiple distributed feature vector representations to achieve event extraction tasks.

**mdBLSTM (bidirectional long short-term memory with a multilevel attention mechanism and dependency-based word embeddings)**

He et al [5] proposed a bidirectional long short-term memory neural network based on a multilevel attention mechanism and dependency-based word embeddings to extract biomedical events.

**Reinforcement learning+knowledge bases**

Zhao et al [32] proposed a framework of reinforcement learning with external biomedical knowledge bases for extracting biomedical events.

**DeepEventMine**

Trieu et al [35] proposed an end-to-end neural model. It uses a multioverlapping directed acyclic graph to detect nested biomedical entities, triggers, roles, and events.

**Hierarchical artificial neural network**

Zhao et al [36] proposed a 2-level modeling method for document-level joint biomedical event extraction.

Table 4 illustrates the overall performance against the state-of-the-art methods with gold standard entities. As seen in this table, our CPJE model achieved only a slight improvement in the trigger recognition task. For the event extraction task, the F_1_ score was significantly better than the other baselines. Notably, the gap between the precision and recall of our model was much smaller than that of the mdBLSTM (bidirectional long short-term memory with a multilevel attention mechanism and dependency-based word embeddings) model, and the precision was much better than that of the RL+KBs model. This indicates that our model had a better effect on reducing cascading errors than the pipeline models. In addition, the hierarchical artificial neural network (HANN) model was also a joint extraction model; however, its performance is disappointing. This is because the HANN model focuses on extracting document-level biomedical events, which contain many cross-sentence entities, triggers, and events. However, other models aim to extract sentence-level events; therefore, the performance of these models is better than that of the HANN model.

**Table 4:** Overall performance on multilevel event extraction compared with the state-of-the-art methods with gold standard entities.

Method	Trigger recognition (%)				Event extraction (%)		
	Precision	Recall	F_1_ score	Precision		Recall	F_1_ score
EventMine^a^	70.79	81.69	75.84	62.28		49.56	55.20
SSL^a,b^	72.17	82.26	76.89	55.76		59.16	57.41
CNN^a,c^	80.92	75.23	77.97	60.56		56.23	58.31
mdBLSTM^a,d^	82.79	76.56	79.55	90.24		44.50	59.61
RL^e^+KBs^a,f^	N/A^g^	N/A	N/A	63.78		56.81	60.09
DeepEventMine^h^	N/A	N/A	N/A	69.91		55.49	61.87
HANN^h,i^	N/A	N/A	N/A	63.91		56.08	59.74
Our model^h^	82.20	78.25	80.18	72.26		55.23	62.80^j^

^a^Pipeline model.

^b^SSL: semisupervised learning.

^c^CNN: convolutional neural network.

^d^mdBLSTM: bidirectional long short-term memory with a multilevel attention mechanism and dependency-based word embeddings

^e^RL: reinforcement learning.

^f^KB: knowledge base

^g^N/A: not applicable.

^h^Joint model.

^i^HANN: hierarchical artificial neural network.

^j^The best value compared with baselines.

### The Performance for Nested Events on MLEE

To evaluate the effectiveness of our model for improving the nested biomedical event extraction, we split the test set into 2 parts (*simple* and *nested*). *Simple* means that 1 event only regards the entities as its arguments; *nested* means that one of the arguments of an event may be another event. In general, nested events are present in regulation, positive regulation, negative regulation, and planned process events.

Table 5 illustrates the performance (F_1_ scores) of the CNN model [3], the RL+KBs model [32], the DeepEventMine [35] model, the HANN [36] model, and our model in the trigger recognition and event extraction subtasks. In the *simple* and *nested* data of triggers, our framework was 0.44% and 1.25% better than the CNN model, which demonstrates that our model can improve the performance of trigger recognition. However, there is no significant difference between simple and nested triggers. In the *nested* data of events, our model was 6.97% higher than the CNN model, 2.57% higher than the RL+KBs model, 9.53% higher than the DeepEventMine model, and 15.8% higher than the HANN model, which illustrates that our CPJE model of using a gate GCN and an attention mechanism helps to enhance the performance of extracting nested events.

**Table 5:** The F1 score performance on simple events, nested events, and all events on the multilevel event extraction corpus.

Subtask and model			Simple (%)		Nested (%)		All (%)
**Trigger**							
	CNN^a^	79.52		78.80		78.52	
	RL^b^+KBs^c^	N/A^d^		N/A		N/A	
	DeepEventMine	N/A		79.12		N/A	
	HANN^e^	N/A		N/A		N/A	
	Our model	79.96^f^		80.05^f^		80.18^f^	
**Event**							
	CNN	61.33		54.29		58.87	
	RL+KBs	N/A		58.69		60.09	
	DeepEventMine	N/A		51.73		61.87	
	HANN	77.08^f^		45.46		59.74	
	Our model	64.85		61.26^f^		62.80^f^	

^a^CNN: convolutional neural network.

^b^RL: reinforcement learning.

^c^KB: knowledge base.

^d^N/A: not applicable.

^e^HANN: hierarchical artificial neural network.

^f^The best value compared with other models.

### The Performance for All Events on MLEE

To illustrate the impact of our framework on different events in more detail, Table 6 presents the event extraction performance for all event types. From this table, we obtain the best extraction performance for dephosphorylation events and the worst performance for transcription events. In addition, the catabolic events had the best extraction precision, and the phosphorylation events had the best extraction recall rate.

**Table 6:** The extraction performance for different events on multilevel event extraction corpus.

Events	Precision (%)	Recall (%)	F_1_ score (%)
Cell proliferation	62.50	58.57	60.47
Development	51.82	66.43	58.22
Blood vessel development	90.42	72.66	80.57
Growth	78.02	50.58	61.37
Death	79.12	44.32	56.81
Breakdown	71.30	48.30	57.59
Remodeling	85.71	58.32	69.41
Synthesis	48.00	20.30	28.53
Gene expression	74.72	82.42	78.38
Transcription	16.67	33.33	22.22
Catabolism	100.00	50.00	66.67
Phosphorylation	90.00	100.00	94.74
Dephosphorylation	100.00	100.00	100.00
Localization	76.86	49.98	60.57
Binding	74.52	51.23	60.71
Regulation	63.82	51.49	56.99
Positive regulation	78.28	50.66	61.51
Negative regulation	64.35	54.69	59.13
Planned process	69.57	51.86	59.42
All	64.85	61.26	62.80

### Overall Performance on BioNLP-ST 2011 GE

To improve persuasion, we extended our experiment to the BioNLP-ST 2011 GE corpus. We compared our event extraction results with those of previous systems using the same corpus, as shown in Table 7. Among them, the Turku Event Extraction System (TEES) [42], EventMine [6], and stacked generalization [25] systems are based on support vector machines with designed features. The TEES-CNNs [4] are CNNs integrated into the TEES system to extract relations and events. The DeepEventMine [35] is based on bidirectional transformers and an overlapping directed acyclic graph to jointly extract biomedical events. The HANN [36] model relies on the GCN and hypergraph to obtain local and global contexts. The KB-driven tree LSTM [33] depends on KB concept embedding to improve the pretrained distributed word representations. The Graph Edge-conditioned Attention Networks with Science BERT (GEANet-SciBERT) [34] adopts a hierarchical graph representation encoded by graph edge-conditioned attention networks to incorporate domain knowledge from the Unified Medical Language System into a pretrained language model. Table 7 illustrates that except for the DeepEventMine, our approach outperformed all previous methods.

**Table 7:** The performance of biomedical event extraction on the BioNLP shared task 2011 Genia event corpus.

Method and event type		Precision (%)	Recall (%)	F_1_ score (%)
**TEES^a,b^**				
	Event total^c^	57.65	49.56	53.30
**EventMine^a^**				
	Event total	63.48	53.35	57.98
**Stacked generalization^a^**				
	Event total	66.46	48.96	56.38
**TEES-CNNs^a,d^**				
	Event total	69.45	49.94	58.07
**HANN^e,f^**				
	Event total	71.73	53.21	61.10
**KB^g^-driven tree LSTM^e,h^**				
	Simple total^i^	85.95	72.62	78.73
	Binding	53.16	37.68	44.10
	Regulation total^j^	55.73	41.73	47.72
	Event total	67.10	52.14	58.65
**GEANet-SciBERT^e,k^**				
	Regulation total	55.21	47.23	50.91
	Event total	64.61	56.11	60.06
**DeepEventMine^e^**				
	Regulation total	62.36	51.88	56.64^l^
	Event total	76.28	55.06	63.96^l^
**Our model^e^**				
	Simple total	82.23	78.88	80.52
	Binding	55.12	37.48	44.62
	Regulation total	57.82	46.39	51.48
	Event total	72.62	53.33	61.50

^a^Pipeline model.

^b^TEES: Turku Event Extraction System.

^c^Represents the overall performance on the test set.

^d^CNN: convolutional neural network.

^e^Joint model.

^f^HANN: hierarchical artificial neural network.

^g^KB: knowledge base.

^h^LSTM: long short-term memory.

^i^Represents the overall performance for simple events on the test set.

^j^Represents the overall performance for nested events on the test set (including regulation, positive regulation, and negative regulation subevents).

^k^GEANet-SciBERT: Graph Edge-conditioned Attention Networks with Science BERT.

^l^The best value compared with other models.

The KB-driven tree LSTM and GEANet-SciBERT both draw on the KB to enhance the semantic representation of words to improve the extraction performance of nested (regulation) events. However, the KB-driven tree LSTM only leverages traditional static word embedding, which cannot deeply integrate information from the KB; thus, its performance on nested events is unsatisfactory.

Unlike the KB-driven tree LSTM method, the GEANet-SciBERT model uses a specialized medical KB and scientific information to enrich the dynamic semantic representation of Bidirectional Encoder Representation from Transformers (BERT) and enhances the capability of inferring nested events via a novel GNN. Thus, the F_1_ scores for the nested event extraction were significantly boosted.

Interestingly, the DeepEventMine had an outstanding performance for extracting nested biomedical events on BioNLP-ST 2011 GE but had a passive performance on MLEE. There are three reasons for this fact. First, the DeepEventMine model jointly learns 4 biomedical information tasks (entity detection, trigger detection, role detection, and event detection), which can share more biomedical features and knowledge when model training. Second, the DeepEventMine model uses a more complex graph structure (multiple overlapping directed acyclic graphs) to obtain rich syntactic information. (Finally, the BioNLP-ST 2011 GE data set size is larger than that of the MLEE data set; thus, the DeepEventMine model can be fully trained on a large corpus and enhance the performance of extracting nested events.

## Discussion

In this section, we will study and discuss the performance of our CPJE model using the MLEE corpus.

### Ablation Study

#### The Impact of the BiLSTM

Although the output of BioBERT contains rich semantic information, it has some noise impact on semantic information after concatenating POS embedding, entity embedding, and BioBERT embedding. In addition, the dimension of the BioBERT output is 768, and the total size after concatenation is more extensive, which tends to cause the phenomenon of combination explosion in the feature space. Therefore, we considered using a BiLSTM, which reduces the total dimension and integrates other information with the BioBERT information to obtain a richer semantic representation.

If we remove the BiLSTM layer, the trigger recognition precision is dropped from 82.20% to 75.64%, and the trigger recognition F_1_ score is dropped from 80.18% to 76.39%, which further affects the event extraction performance (the event extraction F_1_ score is fell from 62.80% to 58.02%).

#### The Impact of Softmax Probability

To evaluate the contribution of the softmax probability distribution after trigger prediction to the event extraction task, we used the traditional joint extraction method (as shown in equation 10), which only uses source information when extracting candidate trigger vectors and event argument vectors.

If we only use the source information (soft trigger) for joint extraction, the event extraction task lacks the probability distribution information after trigger recognition, which results in a decline in the recall rate of the model and further affects the F_1_ scores (the event extraction F_1_ score is dropped from 62.80% to 60.09%). However, the overall result is still slightly higher than the pipeline baseline, which also reflects that joint extraction can eliminate cascading errors.

#### The Impact of GCN

We removed the syntactic structure to evaluate the importance of the GCN network; therefore, the GCN module was useless in our model. If the model lacks the GCN component, the performance of trigger recognition is slightly degraded (the trigger recognition F_1_ score is fell from 80.18% to 78.78%), and the result of event extraction is significantly worse than that of the proposed model (the event extraction F_1_ score is fell from 62.80% to 58.40%).

As the syntactic structure can provide significant potential information for event extraction, the GCN model can be aware of the direction of information flow in syntactic structures and capture these features effectively. Therefore, the GCN model is vital for event extraction.

#### The Impact of Dice Loss

In the face of an imbalance in biomedical corpora, we used the Dice loss function. To verify that the Dice loss function had a better effect on event extraction, we used the cross-entropy loss function for comparison.

A significantly large number of negative examples in the data set indicates that easy-negative examples are extensive. A large number of straightforward examples overwhelmed the training, making the model insufficient to distinguish between positive and hard-negative examples. As the cross-entropy loss is accuracy oriented and each instance contributes equally to the loss function, the precision of the model increases (the event extraction precision is risen from 72.26% to 89.26%), but the F_1_ scores do not increase (the event extraction F_1_ score is dropped from 62.60% to 60.30%). Dice loss is a muted version of the F_1_ score—the harmonic mean of precision and recall. When the positive and negative examples in the data set are unbalanced, the Dice loss will reduce the focus on the easy-negative sample and increase the attention on positive and hard-negative samples, thereby balancing the precision and recall values and increasing the F_1_ scores.

### Visualization

**Figure 3:**
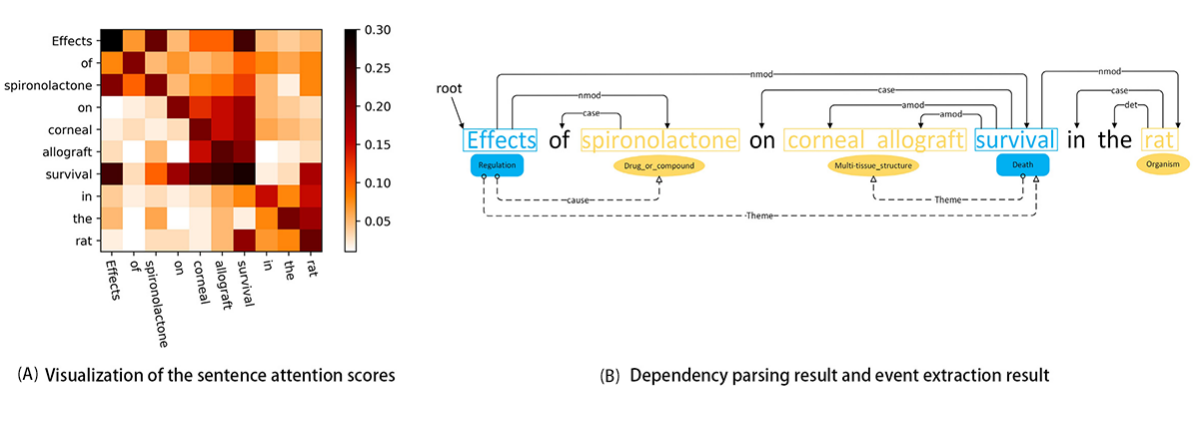
An example of attention-based gate graph neural network effectiveness. (A) Row-wise heap map, where each row is an array of average scores of the 2 heads obtained from the multi-head attention mechanism. The darker the color, the higher the score and the stronger the interaction. (B) Dependency parsing result produced by Stanford CoreNLP and the golden relationships between event triggers and arguments, where yellow boxes represent entity type, and the blue boxes represent event type.

For the effectiveness of the attention-based gate GCN, we used the sentence “Effects of spironolactone on corneal allograft survival in the rat” in Figure 3 as an example to illustrate the captured interaction features. From Figure 3B, we know this sentence contains 2 events: a *regulation* event caused by *effects* and a *death* event caused by *survival*. In addition, a death event is one of the arguments for the regulation event.

As we can see in Figure 3A, the *effects* row has moderately strong links with *Effects* (self), spironolactone (its argument), and *survival* (its argument and another event). Meanwhile, the *survival* row has strong links with *survival* (self), *effects* (another event), and *corneal allograft* (its argument). In addition, the words *rat* and *on* also have strong connections with *survival*, which means that the syntactic dependency information generated by parsing is propagated through the GCN.

### Case Study

#### Overview

Our framework has not achieved state-of-the-art results for the BioNLP-ST 2011 GE corpus. However, the performance of extracting nested biomedical events is satisfactory, particularly in the MLEE corpus. To more intuitively demonstrate the performance of our model in extracting nested biomedical events, we analyzed 3 examples of nested events selected from the MLEE test set to study the strengths and weaknesses of our model compared with the CNN [3].

#### Case 1

**Figure 4:**
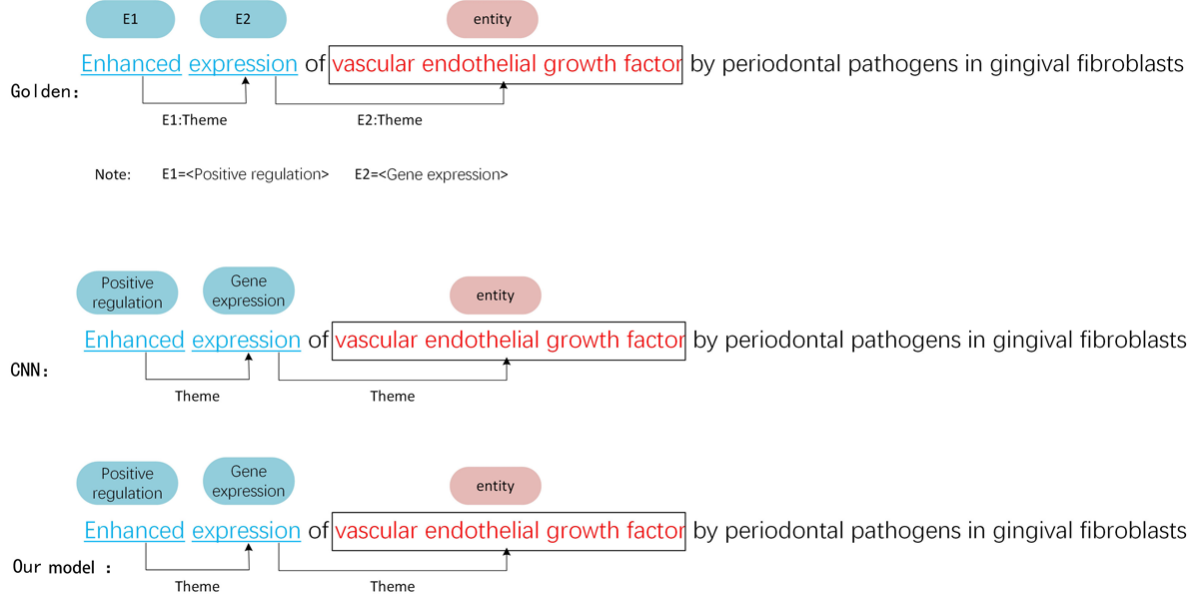
Case study for a simple nested event on the multilevel event extraction corpus. CNN: convolutional neural network.

As shown in Figure 4, case 1 is a simple nested event, where the role type of event argument is only the *theme*. It is a nested event; however, both the CNN and our model obtained correct event extraction results. This is because this sentence does not have a complete component, and perhaps, it is only a part of a complete sentence. The simpler the sentence structure is, the easier it is for the model to extract practical features. Therefore, the extraction performance for such nested events is generally favorable.

#### Case 2

**Figure 5:**
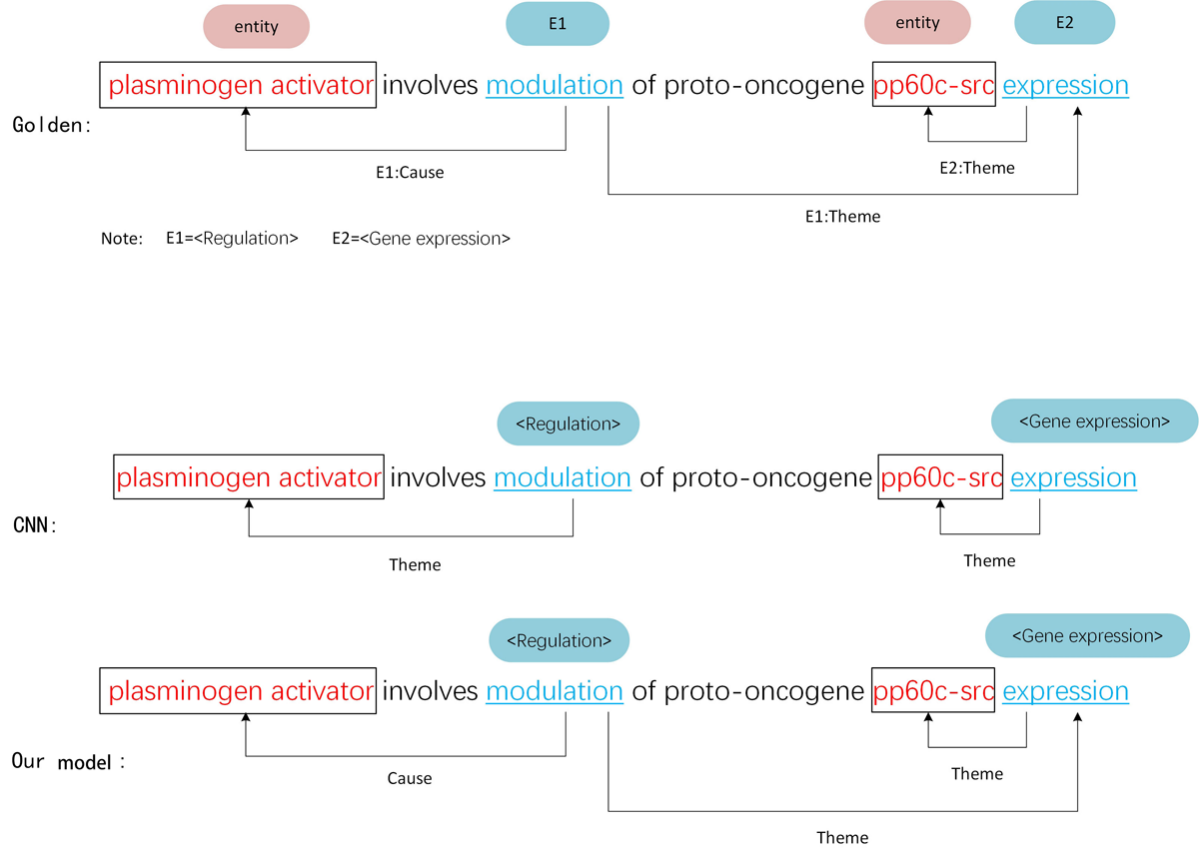
Case study for a common nested event on multilevel event extraction corpus. CNN: convolutional neural network.

Case 2 is a general nested event whose sentence component is complete, and the role types of event arguments are *theme* and *cause*. As shown in Figure 5, the CNN model detects all correct event triggers but cannot detect the correct event arguments. The CNN model is a pipeline approach that considers trigger recognition and argument detection tasks in a cascade rather than a parallel relationship. In general, they first input the text into the CNN model to identify the triggers in the sentence. Then, they construct <trigger, entity> or <trigger, trigger> candidate pairs and input them into the CNN model again to detect the arguments. Finally, rule-based or machine learning-based methods are used to postprocess triggers and arguments to construct complete biomedical events. If there is an error in some of these steps, it will directly affect the performance of event extraction. However, our joint method regards trigger recognition and argument detection as parallel tasks that can provide valid information. Thus, we trained both tasks jointly with one model, and errors could only be generated during the model training.

#### Case 3

**Figure 6:**
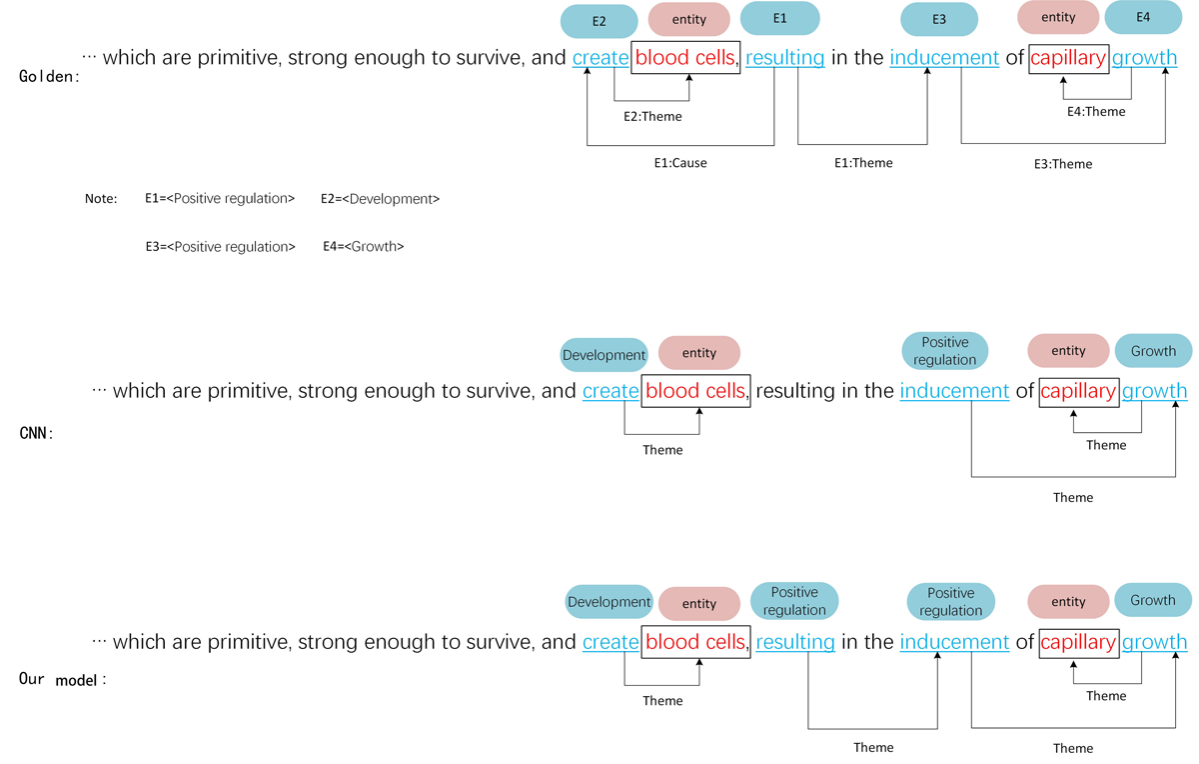
Case study for an across-sentence nested event on multilevel event extraction corpus. CNN: convolutional neural network.

Case 3 is a cross-sentence nested event, as shown in Figure 6. From this example, we can determine what needs to be improved. As multiple events are nested in each other, and some of these events are not in the same sentence, this prevents the model from extracting all events efficiently and accurately. Compared with the CNN model, although our model can identify the *positive regulation* event triggered by *resulting*, it is not in the same clause as the *development* event triggered by *create*, which causes the *positive regulation* event to lack an event argument.

### Conclusions

In this study, a CPJE framework based on a multi-head attention graph CNN is proposed to achieve biomedical event extraction tasks. The cascading errors between the 2 subtasks were reduced because of the use of the joint extraction framework. With the help of the attention-based gate GCN, syntactic dependency information and the interrelations between triggers and related entities were effectively learned; thus, the extraction performance of nested biomedical events improved. The Dice loss replaced the cross-entropy loss, which weakened the negative impact of the imbalanced data set. Overall, the model obtained the best F_1_ score in the MLEE biomedical event extraction corpus and achieved favorable performance on the BioNLP-ST 2011 GE corpus. In the future, we will consider integrating external resource knowledge to allow the model to learn richer information and improve the performance of cross-sentence nested events.

## Abbreviations

BERT: Bidirectional Encoder Representation From Transformers
BiLSTM: bidirectional long short-term memory
BioBERT: Biomedical Bidirectional Encoder Representation From Transformers
BioNLP: biomedical natural language processing
BioNLP-ST: biomedical natural language processing shared task
CNN: convolutional neural network
CPJE: conditional probability joint extraction
GCN: graph convolutional network
GE: Genia event
GEANet-SciBERT: Graph Edge-conditioned Attention Networks with Science BERT
GNN: graph neural network
HANN: hierarchical artificial neural network
KB: knowledge base
LSTM: long short-term memory
mdBLSTM: bidirectional long short-term memory with a multilevel attention mechanism and dependency-based word embeddings
MLEE: multilevel event extraction
POS: parts of speech
RL: reinforcement learning
SGD: stochastic gradient descent
TEES: Turku Event Extraction System

## References

[1] McDonald RT, Pereira FC, Kulick SN, Winters R, Jin Y, White PS (2005). Simple algorithms for complex relation extraction with applications to biomedical IE. Proceedings of the 43rd Annual Meeting on Association for Computational Linguistics.

[2] Kilicoglu H, Bergler S (2011). Effective bio-event extraction using trigger words and syntactic dependencies. Comput Intell.

[3] Wang A, Wang J, Lin H, Zhang J, Yang Z, Xu K (2017). A multiple distributed representation method based on neural network for biomedical event extraction. BMC Med Inform Decis Mak.

[4] Björne J, Salakoski T (2018). Biomedical event extraction using convolutional neural networks and dependency parsing. Proceedings of the BioNLP 2018 workshop.

[5] He X, Li L, Song X, Huang D, Ren F (2019). Multi-level attention based BLSTM neural network for biomedical event extraction. IEICE Trans Inf Syst.

[6] Pyysalo S, Ohta T, Miwa M, Cho H, Tsujii J, Ananiadou S (2012). Event extraction across multiple levels of biological organization. Bioinformatics.

[7] Kim JD, Wang Y, Takagi T, Yonezawa A (2011). Overview of Genia event task in BioNLP shared task 2011. Proceedings of BioNLP Shared Task 2011 Workshop.

[8] Zheng S, Hao Y, Lu D, Bao H, Xu J, Hao H, Xu B (2017). Joint entity and relation extraction based on a hybrid neural network. Neurocomputing.

[9] Ye ZX, Ling ZH (2019). Distant supervision relation extraction with intra-bag and inter-bag attentions. Proceedings of the 2019 Conference of the North American Chapter of the Association for Computational Linguistics: Human Language Technologies.

[10] Feng J, Huang M, Zhao L, Yang Y, Zhu X (2018). Reinforcement learning for relation classification from noisy data. Proceedings of the 32nd AAAI Conference on Artificial Intelligence.

[11] Fu TJ, Li PH, Ma WY (2019). Graphrel: Modeling text as relational graphs for joint entity and relation extraction. Proceedings of the 57th Annual Meeting of the Association for Computational Linguistics.

[12] Guo Z, Zhang Y, Lu W (2019). Attention guided graph convolutional networks for relation extraction. Proceedings of the 57th Annual Meeting of the Association for Computational Linguistics.

[13] Li Q, Ji H, Huang L (2013). Joint event extraction via structured prediction with global features. Proceedings of the 51st Annual Meeting of the Association for Computational Linguistics.

[14] Keith KA, Handler A, Pinkham M, Magliozzi C, McDuffie J, O'Connor B (2017). Identifying civilians killed by police with distantly supervised entity-event extraction. Proceedings of the 2017 Conference on Empirical Methods in Natural Language Processing.

[15] Reichart R, Barzilay R (2012). Multi-event extraction guided by global constraints. Proceedings of the 2012 Conference of the North American Chapter of the Association for Computational Linguistics: Human Language Technologies.

[16] Lu W, Roth D (2012). Automatic event extraction with structured preference modeling. Proceedings of the 50th Annual Meeting of the Association for Computational Linguistics.

[17] Sha L, Qian F, Chang B, Sui Z (2018). Jointly extracting event triggers and arguments by dependency-bridge RNN and tensor-based argument interaction. Proceedings of the 32nd AAAI Conference on Artificial Intelligence.

[18] Nguyen TH, Grishman R (2016). Modeling skip-grams for event detection with convolutional neural networks. Proceedings of the 2016 Conference on Empirical Methods in Natural Language Processing.

[19] Liu X, Luo Z, Huang H (2018). Jointly multiple events extraction via attention-based graph information aggregation. Proceedings of the 2018 Conference on Empirical Methods in Natural Language Processing.

[20] Kim JD, Ohta T, Pyysalo S, Kano Y, Tsujii J (2009). Overview of BioNLP'09 shared task on event extraction. Proceedings of the Workshop on Current Trends in Biomedical Natural Language Processing: Shared Task.

[21] Bossy R, Golik W, Ratkovic Z, Bessières P, Nédellec C (2013). Bionlp shared task 2013 - an overview of the bacteria biotope task. Proceedings of the BioNLP Shared Task 2013 Workshop.

[22] Miwa M, Saetre R, Kim JD, Tsujii J (2010). Event extraction with complex event classification using rich features. J Bioinform Comput Biol.

[23] Miwa M, Thompson P, Ananiadou S (2012). Boosting automatic event extraction from the literature using domain adaptation and coreference resolution. Bioinformatics.

[24] Björne J, Salakoski T (2013). TEES 2.1: automated annotation scheme learning in the BioNLP 2013 shared task. Proceedings of the BioNLP Shared Task 2013 Workshop.

[25] Majumder A, Ekbal A, Naskar SK (2016). Biomolecular event extraction using a stacked generalization-based classifier. Proceedings of the 13th International Conference on Natural Language Processing.

[26] Riedel S, McCallum A (2011). Robust biomedical event extraction with dual decomposition and minimal domain adaptation. Proceedings of BioNLP Shared Task 2011 Workshop.

[27] Venugopal D, Chen C, Gogate V, Ng V (2014). Relieving the Computational Bottleneck: joint inference for event extraction with high-dimensional features. Proceedings of the 2014 Conference on Empirical Methods in Natural Language Processing.

[28] Nguyen DQ, Verspoor K (2019). From POS tagging to dependency parsing for biomedical event extraction. BMC Bioinformatics.

[29] Zhou D, Zhong D (2015). A semi-supervised learning framework for biomedical event extraction based on hidden topics. Artif Intell Med.

[30] Rao S, Marcu D, Knight K, Daumé III H (2017). Biomedical event extraction using abstract meaning representation. Proceedings of the BioNLP 2017 workshop.

[31] Yan S, Wong KC (2020). Context awareness and embedding for biomedical event extraction. Bioinformatics.

[32] Zhao W, Zhao Y, Jiang X, He T, Liu F, Li N (2020). A novel method for multiple biomedical events extraction with reinforcement learning and knowledge bases. Proceedings of the 2020 IEEE International Conference on Bioinformatics and Biomedicine.

[33] Li D, Huang L, Ji H, Han J (2019). Biomedical event extraction based on knowledge-driven tree-LSTM. Proceedings of the 2019 Conference of the North American Chapter of the Association for Computational Linguistics: Human Language Technologies.

[34] Huang KH, Yang M, Peng N (2020). Biomedical event extraction with hierarchical knowledge graphs. Proceedings of the 2020 Conference on Empirical Methods in Natural Language Processing.

[35] Trieu HL, Tran TT, Duong KN, Nguyen A, Miwa M, Ananiadou S (2020). DeepEventMine: end-to-end neural nested event extraction from biomedical texts. Bioinformatics.

[36] Zhao W, Zhang J, Yang J, He T, Ma H, Li Z (2021). A novel joint biomedical event extraction framework via two-level modeling of documents. Inf Sci.

[37] Ramponi A, van der Goot R, Lombardo R, Plank B (2020). Biomedical event extraction as sequence labeling. Proceedings of the 2020 Conference on Empirical Methods in Natural Language Processing.

[38] Lee J, Yoon W, Kim S, Kim D, Kim S, So CH, Kang J (2020). BioBERT: a pre-trained biomedical language representation model for biomedical text mining. Bioinformatics.

[39] Klein D, Manning CD (2003). Accurate unlexicalized parsing. Proceedings of the 41st Annual Meeting of the Association for Computational Linguistics.

[40] Vaswani A, Shazeer N, Parmar N, Uszkoreit J, Jones L, Gomez AN, Kaiser Ł, Polosukhin I (2017). Attention is all you need. Proceedings of Annual Conference on Advances in Neural Information Processing Systems.

[41] Li X, Sun X, Meng Y, Liang J, Wu F, Li J (2020). Dice loss for data-imbalanced NLP tasks. Proceedings of the 58th Annual Meeting of the Association for Computational Linguistics.

[42] Björne J, Salakoski T (2011). Generalizing biomedical event extraction. Proceedings of BioNLP Shared Task 2011 Workshop.

